# Trends in the administration of COVID-19 vaccines with other vaccines in the United States reported to V-safe during December 14, 2020—May 19, 2023

**DOI:** 10.1080/21645515.2024.2361946

**Published:** 2024-06-07

**Authors:** Casey E. Parker, Anne M. Hause, Paige Marquez, Bicheng Zhang, Tanya R. Myers, David K. Shay

**Affiliations:** a Immunization Safety Office, Division of Healthcare Quality Promotion, National Center for Emerging and Zoonotic Infectious Diseases, Centers for Disease Control and Prevention, Atlanta, GA, USA; b ORISE Health Studies Program, Oak Ridge Associated Universities, Oak Ridge, TN, USA

**Keywords:** COVID-19 vaccine, influenza vaccine, co-administration, V-safe

## Abstract

Introduction COVID-19 vaccines may be administered with other vaccines during the same healthcare visit. COVID-19 monovalent (Fall 2021) and bivalent (Fall 2022) vaccine recommendations coincided with annual seasonal influenza vaccination. Data describing the frequency of the co-administration of COVID-19 vaccines with other vaccines are limited. Methods We used V-safe, a voluntary smartphone-based U.S. safety surveillance system established by the CDC, to describe trends in the administration of COVID-19 vaccines with other vaccines reported to V-safe during December 14, 2020 – May 19, 2023. Results Of the 21 million COVID-19 vaccinations reported to V-safe, 2.2% (459,817) were administered with at least 1 other vaccine. Co-administration most frequently occurred during the first week of October 2023 (27,092; 44.1%). Most reports of co-administration included influenza vaccine (393,003; 85.5%). Co-administration was most frequently reported for registrants aged 6 months-6 years (4,872; 4.4%). Conclusion Reports of co-administration to V-safe peaked during October 2023, when influenza vaccination most often occurs, possibly reflecting increased opportunities for multiple vaccinations and greater acceptability of the co-administration of COVID-19 vaccine with other vaccines, especially influenza vaccine.

## Introduction

Shortly after Emergency Use Authorizations (EUAs) were issued for the first COVID-19 vaccines in December 2020, the Centers for Disease Control and Prevention’s (CDC) Advisory Committee on Immunization Practices (ACIP) recommended that COVID-19 vaccines be administered alone (i.e., without other vaccines). A minimum interval of 14 days was recommended between the administration of other vaccines, given the lack of data concerning the co-administration of COVID-19 vaccines with other vaccines.^1^ In May 2021, ACIP updated these recommendations based on accumulated experience, stating that the COVID-19 vaccines may be administered without regard for non-COVID-19 vaccination timing.^2^

On September 23, 2021, ACIP recommended Pfizer-BioNTech COVID-19 monovalent booster doses for persons aged 18 years and older; booster doses of Moderna and Janssen vaccines were recommended on October 21, 2021.^3^ On September 1, 2022, COVID-19 bivalent booster doses were recommended for persons aged 12 years and older, and on October 12, 2022, Pfizer-BioNTech bivalent doses were recommended for children aged 5–11 years.^4^ These recommendations suggested that the peak in COVID-19 vaccination would coincide with the peak in annual seasonal influenza vaccination, which generally occurs during September and October.

Data describing the frequency of co-administration of COVID-19 vaccines with other vaccines are limited. We utilized V-safe data to estimate how the frequency of co-administration reflects updates to vaccine recommendations during December 2020–May 2023.

## Methods

### V-safe

V-safe is a voluntary smartphone-based vaccine safety surveillance system established by CDC in December 2020 to collect data on reactions and health impacts for registrants reporting COVID-19 vaccination.^5^ Following vaccination, persons registered themselves or their dependents with V-safe and self-reported information related to vaccination. Information collected included the registrant’s age, sex, race, ethnicity, date of vaccination, COVID-19 vaccine manufacturer, dose number in vaccine series, and any vaccination(s) administered during the same healthcare visit.

V-safe COVID-19 vaccination data collection began on December 14, 2020, and concluded on May 19, 2023. From December 2020 through June 18, 2021, co-administration reports were retrospectively obtained by V-safe. On June 19, 2021, CDC added an item to the V-safe survey to capture if another vaccine had been received on the same day as a COVID-19 vaccine. Registrants answered “yes” or “no” to the item. A one-time survey was administered from October 28, 2021, through November 28, 2021, to retrospectively collect vaccine type(s) received among registrants reporting co-administration from June 19, 2021, through November 21, 2021. On November 22, 2021, V-safe began prospectively collecting the type of vaccine administered during the same visit. Options for vaccine type received were age-dependent; more than one vaccine could be selected.

### Data analysis

Using the total number of COVID-19 vaccinations reported to V-safe as the denominator, we described vaccine co-administration by demographic characteristics, vaccine type(s) received, and trends over time during three influenza seasons (2020–21, 2021–22, and 2022–23).^6^ The reporting period for each influenza season began on Morbidity and Mortality Weekly Report (MMWR) week 40 and ended week 39 of the following year.^7^ We used SAS software, version 9.4 (SAS Institute Inc), to conduct analyses.

Reports were excluded if the COVID-19 vaccine manufacturer was listed as unknown (*n* = 13,051) or reported age at the time of vaccination did not meet EUA requirements for the reported vaccine received (*n* = 6,052) to limit inaccurate vaccination reports.

### Human subjects

This activity was reviewed by the CDC and was conducted consistent with applicable federal law and CDC policy. See 45 C.F.R. part 46, 21 C.F.R. part 56; 42 U.S.C. §241(d); 5 U.S.C. §552a; 44 U.S.C. §3501 et seq.

## Results

During the study period, V-safe received 21,048,557 reports of COVID-19 vaccination; 2.2% (459,817) indicated co-administration. Over 97% (447,524) of co-administrations were among adults aged ≥18 years (Table 1). Among all age groups, influenza vaccines were the most frequently (85.5%; 393,003) co-administered vaccine (Table 2). Most reports of co-administration indicated only one additional vaccine was received (97.1%; 446,556) (Table 3). Reports of two or more vaccines administered during the same visit were most frequent (27.9%; 1,360) among children aged 6 months-6 years.

**Table 1.** Frequencies of COVID-19 vaccine co-administration among reports (N = 21,048,557) received by V-safe by influenza season^a^.

	2020–21^b^			2021–22^c^			2022–23^d^		
	n = 17,646,243			n = 3,113,574			n = 288,740		
Characteristic	No.	N	%	No.	N	%	No.	N	%
**Sex**									
Female	43,539	11,003,696	0.4	170,111	1,887,983	9.0	60,608	181,271	33.4
Male	30,630	6,481,686	0.5	113,215	1,204,765	9.4	37,832	105,537	35.9
Other^e^	63	20,516	0.3	559	3,664	15.3	149	329	45.3
Unknown	617	140,345	0.4	1,913	17,162	11.2	581	1,603	36.2
All	74,849	17,646,243	0.4	285,798	3,113,574	9.2	99,170	288,740	34.3
**Age range, years**									
0–6	–	–	–	3,012	102,069	3.0	1,860	9,123	20.4
7–17	1,279	363,206	0.4	4,517	153,253	3.0	1,625	6,229	26.1
18–49	23,616	8,336,002	0.3	108,947	947,018	11.5	33,514	81,475	41.1
50–64	17,632	4,942,696	0.4	84,668	892,068	9.5	31,057	85,922	36.15
≥65	32,322	4,004,339	0.8	84,654	1,019,166	8.3	31,114	105,991	29.4
**Race**									
White	56,035	11,989,123	0.5	230,328	2,313,904	10.0	81,700	228,847	35.7
Black	4,451	1,144,461	0.4	16,837	210,474	8.0	6,066	20,299	29.9
Asian	3,964	1,277,524	0.3	14,518	214,665	6.8	4,700	17,721	26.5
AI/AN	460	104,363	0.4	1,402	14,618	9.6	479	1,281	37.4
NHPI	209	58,415	0.4	596	8,342	7.1	194	622	31.2
Multiracial	1,187	335,331	0.4	6,615	71,934	9.2	2,329	6,756	34.5
Other^e^	2,257	611,017	0.4	5,671	83,831	6.8	1,662	5,487	30.3
Unknown	6,286	2,126,009	0.3	9,831	195,806	5.0	2,040	7,727	26.4
**Ethnicity**									
Hispanic/Latino	6,189	1,813,812	0.3	20,354	264,982	7.7	6,392	19,796	32.3
Not Hispanic or Latino	60,908	13,419,272	0.5	252,688	2,612,301	9.7	89,815	257,872	34.8
Unknown	7,752	2,413,159	0.3	12,756	236,291	5.4	2,963	11,072	26.8
**Manufacturer**									
Pfizer-BioNtech	61,545	9,255,841	0.7	163,012	1,576,757	10.3	58,534	160,423	36.5
Moderna	12,770	7,889,098	0.2	121,478	1,501,842	8.1	40,570	127,783	31.7
Janssen	534	501,304	0.1	1,304	34,678	3.8	53	344	15.4
Novavax	–	–	–	4	297	1.4	13	190	6.8

No: Number of reports indicating a co-administration received by V-safe.

N: Total number of COVID-19 vaccinations reported to V-safe.

AI/AN: American Indian/Alaska Native; NHPI: Native Hawaiian/Pacific Islander.

^a^The reporting period for each Influenza season begins MMWR week 40 and ends week 39 of the following year.

^b^Data collection for the 2020–21 influenza season began on December 14, 2020, during MMWR week 51, and ended on October 2, 2021.

^c^The reporting period for 2021–22 influenza season began on October 3, 2021, and ended on October 1, 2022.

^d^The reporting period for the 2022–23 influenza season began on October 2, 2022; the study period is through MMWR week 20.

^e^Other or prefer not to say.

− Not applicable.

**Table 2.** Type of vaccine(s) administered with a COVID-19 vaccine among reports (N = 21,048,557) received by V-safe.

	6mo-6y		7-17y		18-49y		50-64y		≥65y	
	n = 111,192		n = 522,688		n = 9,364,495		n = 5,920,686		n = 5,129,496	
Type of Vaccine^a^	N	%	N	%	N	%	N	%	N	%
Any	4,872	4.4	7,421	1.4	166,077	1.8	133,357	2.3	148,090	2.9
Influenza	3,294	3.0	5,471	1.1	148,806	1.6	109,422	1.9	126,010	2.5
Hepatitis B	367	0.3	32	<0.1	–	–	–	–	–	–
Rotavirus	374	0.3	–	–	–	–	–	–	–	–
Tetanus	946	0.9	226	<0.1	3,071	<0.1	2,930	0.1	3,102	0.1
Hib	494	0.4	–	–	–	–	–	–	–	–
Pneumonia	355	0.3	–	–	1,010	<0.1	2,924	0.1	7,545	0.2
Hepatitis A	464	0.4	22	<0.1	–	–	–	–	–	–
Polio	481	0.4	11	<0.1	–	–	–	–	–	–
MMR	556	0.5	30	<0.1	–	–	–	–	–	–
Chickenpox	403	0.4	12	<0.1	–	–	–	–	–	–
HPV	–	–	458	0.1	764	<0.1	75	<0.1	43	<0.1
Meningitis	–	–	285	0.1	187	<0.1	93	<0.1	60	<0.1
Shingles	–	–	–	–	203	<0.1	14,412	0.2	7,331	0.1
Monkeypox	–	–	2	<0.1	155	<0.1	99	<0.1	32	<0.1
Missing	54	0.1	1,177	0.2	11,465	0.1	6,387	0.1	7,445	0.2
Unknown	214	0.2	134	<0.1	1,166	<0.1	750	<0.1	776	<0.1
Other	206	0.2	139	<0.1	1,951	<0.1	1,932	<0.1	1,450	<0.1

Hib: Haemophilus influenzae bacteria; MMR: Measles, mumps, and rubella; HPV: human papillomavirus.

^a^Category is not exclusive; more than one vaccine type could be selected by registrant.

− No reports for this type.

**Table 3.** Number of vaccines received in addition to a COVID-19 vaccine received by V-safe.

	6 mo-6y		7-17y		18-49y		50-64y		≥65y		Total	
	n = 4,872		n = 7,421		n = 166,077		n = 133,357		n = 148,090		n = 459,817	
Number of co-administered vaccines	No.	%	No.	%	No.	%	No.	%	No.	%	No.	%
1	3,512	72.1	7,032	94.8	163,753	98.6	128,559	96.4	143,700	97.0	446,556	97.1
2	402	8.3	251	3.4	2,079	1.3	4,106	3.1	3,442	2.3	10,280	2.2
3	394	8.1	110	1.5	176	0.1	565	0.4	642	0.4	1,887	0.4
4	263	5.4	22	0.3	37	<0.1	92	0.1	256	0.2	670	0.2
5	175	3.6	2	<0.1	16	<0.1	25	<0.1	40	<0.1	258	0.1
≥6	126	2.6	4	<0.1	16	<0.1	10	<0.1	10	<0.1	166	<0.1

**Figure 1.**
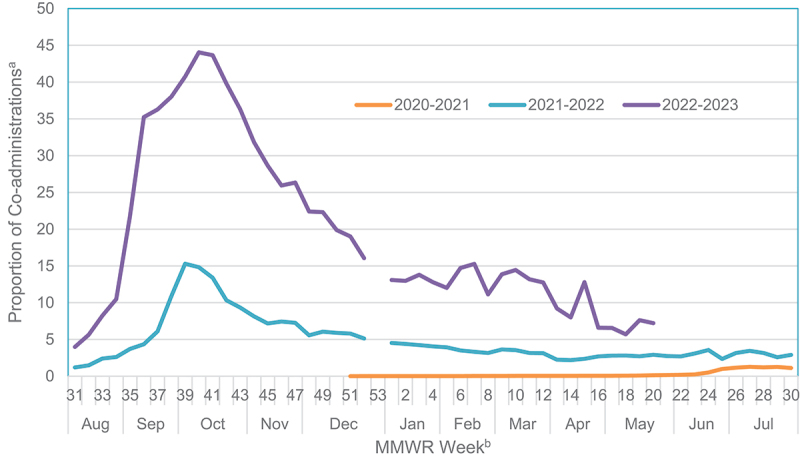
Reports of COVID-19 vaccines co-administered with another vaccine received by V-safe.

^a^Proportion of reports indicating a COVID-19 vaccine co-administration with another vaccine during the same healthcare visit

^b^ The reporting period for each influenza season begins in MMWR week 40 and ends week 39 of the following year. For the reporting period of the 2020-21 influenza season, data collection began on December 14, 2020, during MMWR week 51, and ended on October 2, 2021. The reporting period for the 2021-22 influenza season began on October 3, 2021, and ended on October 1, 2022. The reporting period for the 2022-23 influenza season began on October 2, 2022; the study period is through MMWR week 20.

Prior to May 2021, only 0.04% (5,925) of reported COVID-19 vaccinations indicated co-administration with another vaccine. Following updated ACIP guidance, 8.7% (453,892) of reports indicated co-administration. During the reporting period for the 2020–21 influenza season included in the study, 0.4% (74,849) of reported COVID-19 vaccinations were co-administered with another vaccine. During the 2021–22 season, 9.2% (285,798) of COVID-19 vaccinations were administered with another vaccine. Through the end of the study period during the 2022–23 season, 34.4% (99,170) of COVID-19 vaccinations were administered with another vaccine. Overall, co-administration was most frequently reported in the first week of October 2023 (44.1%; 27092) (Figure 1).

## Discussion

In V-safe, reports of vaccines administered with COVID-19 vaccine were uncommon during the 2020–21 influenza season; co-administration was not recommended until May 2021, after the influenza season. COVID-19 booster vaccine recommendations coincided with the peak timing for annual influenza vaccination, increasing the likelihood for co-administration of COVID-19 vaccines with influenza vaccines. Receipt of multiple vaccines during the 2021–22 and 2022–23 seasons was most common during September and October, when most annual influenza vaccines are administered.^8^ A study describing the administration of bivalent COVID-19 vaccines with influenza vaccines using data from the Vaccine Safety Datalink (VSD) found similar vaccination patterns.^9^

Influenza vaccines were the most common vaccines administered with COVID-19 vaccines in V-safe data. Annual seasonal influenza vaccination is recommended for all persons aged 6 months and older who do not have a contraindication.^10^ The co-administration of influenza vaccines with other vaccines including measles, mumps, and rubella (MMR), varicella, pneumococcal, and tetanus-containing vaccines is safe, based on extensive clinical experience and observational data.^11^ Research has indicated that co-administration of COVID-19 and influenza vaccines is also a safe and effective clinical practice.^12–15^

Over 97% of co-administrations reported to V-safe were from adults aged ≥18 years, reflecting overall trends in COVID-19 vaccine coverage. As of June 2023, 88.0% of adults were estimated to have received at least one COVID-19 dose, compared to 36.8% of children.^16^ However, the receipt of two or more vaccines in addition to a COVID-19 vaccine was most common among children aged 6 months-6 years (28%). This finding reflects the recommended routine childhood immunization schedule, which often includes more than one vaccination at healthcare visits from 2 months to 6 years of age.^17^

Historically, the adult immunization schedule has provided fewer opportunities for co-administration.^18^ Survey results reported by the CDC found relatively high levels (72%) of acceptability of co-administration of influenza vaccines with COVID-19 vaccine among adults.^19^ On June 21, 2023, ACIP recommended that adults aged ≥60 years may receive a single dose of a respiratory syncytial virus (RSV) vaccine.^20^ The addition of COVID-19 and RSV vaccines to the adult immunization schedule suggests education about recommendations for the administration of multiple vaccines during a single healthcare visit could lead to improved vaccine coverage in adult populations.^18^

### Limitations

V-safe is a voluntary program; data might not be representative of the vaccinated population. Recall bias and lack of verification concerning vaccinations received may have resulted in misclassification of vaccination data. Reasons for the increased uptake in co-administration cannot be determined as specific questions were not included in V-safe surveys.

## Conclusions

Reports of administration of COVID-19 and other vaccines to V-safe were highest during the 2022–23 influenza season, possibly reflecting greater acceptability of co-administration of COVID-19 vaccine with other vaccines, especially influenza vaccines.

## Data Availability

Data collected through V-safe from 12/13/2020 to 9/25/2022 is available from https://data.cdc.gov/Public-Health-Surveillance/v-safe-COVID-19/dqgu-gg5d/about_data.
